# Tolerability of trivalent inactivated influenza vaccine among pregnant women, 2015

**DOI:** 10.1186/s12884-018-1712-6

**Published:** 2018-04-23

**Authors:** Suvanna Asavapiriyanont, Wanitchaya Kittikraisak, Piyarat Suntarattiwong, Darunee Ditsungnoen, Surasak Kaoiean, Podjanee Phadungkiatwatana, Nattinee Srisantiroj, Tawee Chotpitayasunondh, Fatimah S. Dawood, Kim A. Lindblade

**Affiliations:** 1 Rajavithi Hospital, Bangkok, Thailand; 2 Influenza Program, Thailand Ministry of Public Health – U.S. Centers for Disease Control and Prevention Collaboration, Nonthaburi, Thailand; 3 Queen Sirikit National Institute of Child Health, Bangkok, Thailand; 4 Influenza Division, U.S. Centers for Disease Control and Prevention, Atlanta, USA

**Keywords:** Tolerability, Influenza, Vaccination, Pregnant women, Thailand

## Abstract

**Background:**

Thailand recommends influenza vaccination among pregnant women. We conducted a cohort study to determine if the prevalence of adverse events following immunization (AEFIs) with influenza vaccine among Thai pregnant women was similar to that often cited among healthy adults.

**Methods:**

Women who were ≥17 gestational weeks and ≥18 years of age were recruited. Demographic and health history data were collected using structured questionnaires. Women were provided with symptom diary, ruler to measure local reaction(s), and thermometer to measure body temperature. AEFIs were defined as any new symptom/abnormality occurring within four weeks after vaccination. The diaries were abstracted for frequency, duration, and level of discomfort/inconvenience of the AEFIs. Serious adverse events (SAEs) and the likelihood of AEFIs being associated with vaccination were determined using standard definitions.

**Results:**

Among 305 women enrolled between July–November 2015, median age was 29 years. Of these, 223 (73%) were in their third trimester, 271 (89%) had completed secondary school or higher, and 20 (7%) reported ≥1 pre-existing conditions. AEFIs were reported in 134 women (44%; 95% confidence interval [CI] 38–50%). Soreness at the injection site (74, 24%; CI 19–29%), general weakness (50, 16%; CI 12–21%), muscle ache (49, 16%; CI 12–21%), and headache (45, 15%; CI 1–19%) were most common. Of those with AEFIs, 120 (89%) reported symptom/abnormality occurred on day 0 or day 1 following vaccination. Ten women (7%) reported the AEFIs affected daily activities. The AEFIs generally spontaneously resolved within 24 h of onset. There were two vaccine-unrelated SAEs. Of 294 women with complete follow-up, 279 (95%) had term deliveries, 12 (4%) had preterm deliveries, and 3 (1%) had miscarriage or stillbirth.

**Conclusion:**

In our cohort, AEFIs with influenza vaccine occurred with similar frequency to those reported among healthy adults in other studies, and were generally mild and self-limited. No influenza vaccine-associated SAEs were identified.

## Background

Pregnant women are at high risk of hospitalization from influenza virus infection, with the highest rates of influenza-associated hospitalization documented in the third trimester [1–5]. Influenza vaccination during pregnancy is safe for both the mother and fetus [6–10], and has been shown to be effective at preventing influenza in pregnant women and among their infants in the first few months of life [11–15]. The World Health Organization (WHO) recommends pregnant women receive seasonal influenza vaccine and suggests that countries looking to initiate or expand their influenza vaccination program prioritize pregnant women [1].

In 2009, the Thailand Ministry of Public Health (MOPH) issued a recommendation that pregnant women should receive influenza vaccine after the third month of pregnancy [2]. However, influenza vaccination coverage among pregnant women remains suboptimal in Thailand. A recent analysis by Owusu et al. revealed very low uptake of influenza vaccine in Thai pregnant women: 1.1% in 2010, 0.7% in 2011, and 0.9% in 2012 [16]. These data indicate that the national policy on influenza vaccination in pregnant women is not widely implemented. While a limited supply of free vaccine may be a contributor to the low coverage rates, the same analysis found significantly higher vaccination rates in persons with chronic disease (9–14%) and those aged 65 years and older (11–20%). The lower vaccination rates in pregnant women could be due to operational issues, but concerns by both pregnant women and their clinicians about the safety of the influenza vaccine during pregnancy may also limit vaccination rates [6].

While data from multiple studies have shown the influenza vaccine to be safe and well-tolerated during pregnancy and for the children aged ≥6 months [7–11, 17, 18], similar data are not available from Thailand. Adverse event following immunization (AEFI) reporting has been included as part of Thailand’s surveillance and investigation system since 2003 in order to improve the safety and performance of the national vaccination program which covers all vaccinations provided in the country [19]. However, the database includes very few pregnant women who received influenza vaccination and relies solely on passive reporting. As a result, the local evidence base for the safety of the influenza vaccine among pregnant women in Thailand remains limited. We conducted a prospective cohort study of Thai women who received influenza vaccination during the second or third trimesters of pregnancy to systematically document the frequency of AEFIs after influenza vaccination and to inform the risk communication messages to overcome vaccine hesitancy among pregnant women.

## Methods

### Setting and study design

The study was conducted at Rajavithi hospital in downtown Bangkok. Rajavithi is a tertiary level, 1200 bed hospital that began as a women’s hospital but is now a full service medical facility. Approximately 150 women are seen for antenatal care (ANC) and 20 women deliver a baby on a daily basis. Among 5185 women with delivery outcomes at Rajavithi hospital in 2015, 80% delivered full term, live infants, 19% delivered preterm infants, and 1% delivered stillborn infants.

We used a cohort study design to enroll women at the time of influenza vaccination (Influvac^®^, Abbott Biologicals B.V., The Netherlands) [20] and follow them through the first four weeks post-vaccination to measure the frequency of AEFIs. We also abstracted delivery outcomes to measure the rate of adverse birth outcomes. There was no contemporaneous control group. However, the frequency of adverse birth outcomes among the study cohort was compared to the general population of women delivering at Rajavithi hospital during the previous year.

In preparation for a separate cohort study of the impact of influenza vaccination on birth outcomes, the hospital strengthened its influenza vaccination program by relocating the influenza vaccination site to the ANC clinic, providing educational seminars to hospital staff, including physicians, on the benefits and safety of the influenza vaccine, and increasing the communication materials posted around the ANC clinic.

### Enrollment criteria

Pregnant women were approached for enrollment if they were ≥17 weeks of pregnancy and ≥18 years of age, prescribed vaccination with the Southern Hemisphere formulation of the inactivated, trivalent influenza vaccine (IIV3) on enrollment day, and were able to read and write in Thai. Pregnant women were excluded if they had received any vaccination in the past four weeks or were scheduled to receive any other vaccine on enrollment day or the next four weeks, had a history of Guillain-Barre syndrome or severe allergic reaction following any vaccination, had an apparent mental disability, or were not given a follow-up ANC appointment during which they would be able to return a study symptom diary.

### Definitions

An AEFI was defined according to Thai national guidelines as any *new* symptom or abnormality occurring within four weeks after influenza vaccination [19]. A serious adverse event (SAE) was defined as any of the following: death, life-threatening illness; hospitalization or prolongation of hospitalization; permanent disability or permanent damage; congenital anomaly or birth defect; any illness requiring intervention to prevent permanent impairment or damage; or any other important medical event [21].

### Data collection

Following written informed consent, study nurses administered a structured questionnaire to participants to collect demographic and health history data, including diagnoses of chronic diseases (asthma, chronic lung disease, chronic heart disease, kidney disease, liver disease, neurologic or neuromuscular disorders, hemoglobinopathy, metabolic disease, immunosuppressive conditions, HIV infection, or cancer). Study nurses instructed participants on how to report new symptoms or abnormalities using a daily diary, how to use a ruler to measure the diameter of redness and induration at the injection site, and how to take their body temperature using an axillary thermometer. Participants were asked to record the presence or absence of specific AEFIs known to be associated with vaccination to describe their perceived level of discomfort and inconvenience from each AEFI on a 3-point scale. The solicited AEFIs included soreness/pain and redness at injection site, induration around injection site, fever/feverish, headache, general weakness, muscle ache, nausea, and pruritus. Additionally, the participants were asked to measure body temperature once a day at the same time of day for the first four days after vaccination. Participants were also asked to record hospitalizations for any reason for four weeks following vaccination. Participants were also instructed to record any other new symptoms not specifically requested during the four weeks following vaccination. Participants were asked to return the diary at their subsequent ANC visit. Data from the diary were abstracted into a structured database for analysis.

Following enrollment and vaccination, participants were observed for 30 min, as required by the national guidelines, at the ANC clinic for any AEFIs. On day 3 following vaccination, a study nurse phoned all participants to identify any AEFI requiring medical attention and to ensure that medical care was received. Within a few days of the expected delivery date, participants were phoned to determine whether the delivery had occurred, after which study nurses reviewed medical records to collect data on estimated gestational age and delivery outcomes. The delivery outcomes collected included miscarriage defined as premature loss of a fetus before 24 weeks of pregnancy, stillbirth defined as birth of a fetus from 24 weeks gestation showing no vital signs [22], prematurity defined as live birth at <37 weeks gestation [23], and term delivery defined as live birth at ≥37 weeks), and birth weight. Any hospital visits or hospitalizations were verified using medical records.

### The diary

A diary was used to collect information on the presence/absence of the solicited AEFIs during the first four days, perceived discomfort, the number of days until the AEFI resolved, and AEFI management. Unsolicited AEFIs occurred during the first four days were recorded in a similar fashion as solicited AEFIs, except that they were recorded only when presence. An extra space was available to record any AEFI that occurred from day 5 up to 4 weeks. The following discomfort and inconvenience scale was used: mild (does not interfere with daily activity); moderate (affects daily activity); and severe (prevents daily activity) [24, 25]. AEFI management was self-reported, verified by chart review and categorized as requiring no management; over-the-counter medicine only; physician consultation with prescription for pain relief; physician consultation with prescription for pain relief plus other medicines; or hospitalization. The likely causal association between influenza vaccination and any SAE was graded by a study physician according to the WHO's criteria: certain or very likely, probably, possibly, unlikely, unrelated, or unclassifiable [21].

### Sample size

A sample size of 289 was calculated to estimate the prevalence of an AEFI of 25%, with a precision of ±5 percentage-points or an AEFI with a prevalence as low as 5% with a precision of ±3 percentage-points, assuming a Type I error rate of 5%. To account for an expected loss to follow-up of 5%, an enrollment goal of 305 pregnant women was estimated.

## Results

Between July and November 2015, all 378 women with influenza vaccination prescription were screened for enrollment, of whom 308 (81%) were eligible for inclusion. Of those who were eligible, 305 (99%) consented to participate. All participants returned complete diaries, and 294 (96%) had delivery outcome data collected. The median age of participants was 29 years (interquartile range [IQR] 24–34 years), and 271 (89%) had completed secondary school or higher. Most (184, 60%) participants were multiparous, 223 (73%) were in the third trimester (Table 1). Among those who were multiparous, 33 (18%) reported at least one prior influenza vaccination before the current pregnancy. Pre-existing condition was reported by 20 (7%) participants. Four participants had allergy, 4 had metabolic disease, 3 had asthma, 2 had hemoglobinopathy, 2 had heart disease, 2 had HIV infection, and one each had liver disease, epilepsy, or depression.

**Table 1:** Characteristics of 305 pregnant women enrolled in the cohort

Characteristics at enrollment	Frequency (%)
Age at enrollment (years)	
≤20	17 (6)
>20 to 30	151 (49)
>30 to 40	131 (43)
>40	6 (2)
Gestational age	
Second trimester	83 (27)
Third trimester	222 (73)
Education level	
Some or completed primary school	34 (11)
Completed secondary school	74 (24)
Completed diploma or high vocational school	77 (25)
Completed bachelor degree or higher	120 (39)
First pregnancy	121 (40)
Reported having underlying medical condition	20 (7)
Reported receiving influenza vaccination before this pregnancy^a^	33 (18)

^a^Among those who were multiparous

**Fig. 1:**
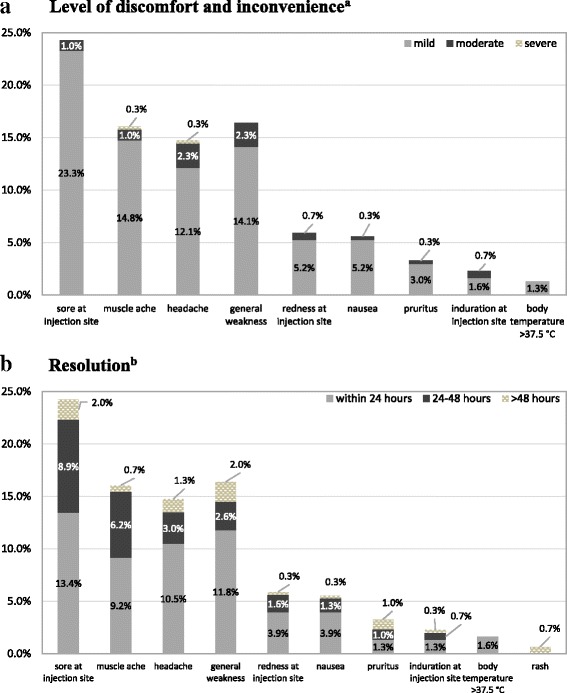
Level of discomfort and inconvenience was self-reported and classified using a 3-point scale: a) mild, did not interfere with daily activity, b) moderate, affected daily activity, and c) severe, prevented daily activity. For redness and induration at/around injection site, the following classification was used: a) mild, reaction at injection of <1 cm in diameter, b) moderate, reaction at injection between 1 and <2 cm in diameter, and c) severe, reaction at injection that is ≥2 cm in diameter. For body temperature, the following classification was used: a) mild, temperature between 37.5 and <39.0 °C, b) moderate, temperature between 39 and <40 °C, and c) severe, temperature ≥40 °C. **a** In addition to the adverse events shown in the fig. 1b, there were one participant reported a fever onset 25 days after vaccination (resolved in 24 hours of onset), two participants who reported having rash (resolved 10 and 13 days following onset), and two participants with vaccine-unrelated serious adverse events (resolution could not be determined). **b** From symptom onset

Overall, 134 (44%; 95% confidence interval [CI] 38–50%) participants reported ≥1 AEFIs during the four-week follow-up (Table 2). Of these, 120 (89%) reported the AEFIs occurred on the day of or one day following vaccination. Almost all AEFIs (132, 99%) occurred within four days following vaccination. The other two AEFIs occurred at 19 and 25 days following vaccination. The most common solicited AEFIs reported by participants were soreness at the injection site (74, 24%; CI 19–29%), general weakness (50, 16%; CI 12–21%), muscle ache (49, 16%; CI 12–21%), and headache (45, 15%; CI 11–19%) (Table 2). Five (2%; CI 0.5–4%) participants reported having fever or feeling feverish, of whom, 4 (1%; CI 0.3–3%) reported having a measured axillary temperature of >37.5 °C starting on day 3 or day 4 while one reported feeling feverish and having the fever onset 25 days following vaccination. The four women with early fever onset had temperatures of <39.0 °C and resolution of fever within 24 h. Temperature was not measured for the participant with late fever onset and she did not seek care. Two participants reported a body rash as an unsolicited AEFI (0.7%; CI 0–2%). Ten women (7%) with AEFIs reported that the AEFIs affected their daily activities (Fig. 1a). All, except three AEFIs, spontaneously resolved. AEFIs were resolved within 24 h of onset in the majority of participants: 36 (72%) of those with general weakness, 32 (71%) of those with headache, 12 (71%) of those with nausea, 12 (67%) of those with redness at injection site, 28 (57%) of those with muscle ache, 4 (57%) of those with induration at injection site, 41 (55%) of those with sore at injection site, and 4 (40%) of those with pruritus. In the two participants with body rash, resolution occurred 10 and 13 days after the onset. Two potential SAEs (decreased fetal movement and chorioamnionitis) were reported during the 4-week follow-up (0.7%; CI 0–2%) but determined to be unrelated to the vaccine by a study physician. No hospitalizations were identified (0%; CI 0–1%).

**Table 2:** Frequency of adverse events occurring within four weeks of influenza vaccination among 305 pregnant women, Bangkok, Thailand^a, b^

Symptoms	Pregnant women, Bangkok		Comparison group 1^d^	Comparison group 2^d^
	Frequency	% (95% confidence interval	%	%
One or more adverse events	134	44 (38.0, 50.0)	NR	NR
Pain/soreness at injection site	74	24.3 (19.4, 29.1)	36.4	1–10
Redness at injection site	18	5.9 (3.2, 8.6)	1.9	1–10
Induration/swelling at injection site	7	2.3 (0.6, 4.0)	2.1	1–10
Body temperature >37.5 °C^c^	4	1.3 (0, 2.6)	1.6	1–10
Headache	45	14.8 (10.8, 18.8)	15.9	1–10
General weakness	50	16.4 (12.4, 21.0)	15.8	1–10
Muscle ache	49	16.1 (11.9, 20.2)	16.4	1–10
Nausea	17	5.6 (3.0, 8.2)	NR	NR
Pruritus	10	3.3 (1.3, 5.3)	NR	NR
Rash	2	0.7 (0, 2.3)	NR	NR

*Abbreviations*: *NR* not reported

^a^All except rash were solicited adverse events

^b^In addition to the adverse events shown in the Table 2, two women had serious adverse events (decreased fetal movement and chorioamniitis) that were judged unrelated to vaccine

^c^Excluding one participant who reported a fever onset 25 days after vaccination whose body temperature was not measured

^d^Comparison group data from Fluarix Quadrivalent package insert [26] (group 1) and Influvac Trivalent package insert [20] (group 2)

There were 294 participants who were interviewed after delivery and had birth outcomes abstracted from the medical chart: 280 (95%; CI 92–97%) delivered full term, live infants; 12 (4%; CI 2–7%) delivered preterm live infants; 1 (0.3%; CI 0–2%) miscarried; and 1 (0.3%; CI 0–2%) had a stillbirth. The median birthweight of term and preterm infants were 3125 g (IQR 2928–3365) and 2521 g (IQR 2168–2851), respectively.

## Discussion

We followed 305 Thai pregnant women prospectively for four weeks after influenza vaccination for solicited and unsolicited AEFIs and collected data on their delivery outcomes. Forty-four percent of women reported one or more AEFIs, but the majority of which were minor and self-limiting, and frequently reported after influenza vaccination in the general population. Fever occurred infrequently among participants, and none of the participants had axillary temperature ≥39.0 °C. None of the women in our study experienced an SAE that was deemed vaccine-related.

The most common types and frequency of AEFIs identified prospectively in this cohort of Thai pregnant women were similar to those reported in clinical trials of inactivated influenza vaccine in non-pregnant adults. Compared with data from Fluarix Quadrivalent package insert, we found similar rates of induration/swelling, fever, headache, weakness, and muscle ache; a lower rate of soreness/pain; and a higher rate of redness (Table 2) [26]. We found similar rates of induration/swelling, fever, and redness, and a higher rates of soreness/pain, weakenss, muscle ache, and headache when compared our findings with data from Influvac Trivalent package insert [20]. Frequency of AEFIs in our study was higher than that reported in some other studies. In both a post-vaccination cluster survey of pregnant women in Laos and a study conducted among pregnant women in Australia, 13% of pregnant women reported experiencing an AEFI [27, 28], although both of these studies followed women for AEFIs only up through seven days after vaccination compared to our month of follow-up, and neither used diary cards.

Although the study was not powered to estimate the frequency of rare events, the prevalence of stillbirth and miscarriage among the women in our study who received IIV3 was lower than that found among all pregnant women delivering at the hospital in the year prior to the study (80% delivered full term, live infants, 19% delivered preterm infants, and 1% delivered stillborn infants).

Several strengths of our study include: a defined cohort with a high rate of follow-up, a standardized diary to collect AEFI data, and characterization of AEFIs using standard definitions. In addition, chart abstraction was performed to collect selected delivery outcomes. However, several limitations should be considered when interpreting our findings. First, assessment of AEFIs was self-reported. Due to the wording on the symptom diary, the study could not differentiate between generalized myalgia and muscle ache at the site of injection and a misclassification between the two may have occurred. Second, the study was not powered to estimate the frequency of rare events and SAEs. Third, the study was designed to collect information only for up to four weeks following vaccination to avoid over burdening pregnant women keeping the diary. However, previous studies have shown that AEFIs from influenza vaccine usually begin soon after vaccination and last 1–2 days [24], so it is likely that the study captured most acute and common events. In addition, because impact of vaccination on delivery outcomes is an important issue in assessing vaccine tolerability and safety in pregnant women, we did collect data on delivery outcomes beyond the four-week follow-up window. Fourth, influenza vaccination history prior to this current pregnancy was self-reported and not verified. Participants may have mistakenly reported other vaccinations received as influenza vaccination; this might apply especially to multiparous participants who received the required tetanus toxoid vaccination during previous pregnancies. Lastly, there was no contemporaneous control group, but the frequency of most common AEFIs was compared to findings from similar studies [19, 20, 26–28] and frequency of adverse delivery outcomes among the study cohort was compared to background frequency among the general population of women delivering at Rajavithi hospital during the previous year.

## Conclusions

We found that influenza vaccine given in the second or third trimester of pregnancy was well tolerated by women in our study. Rates of reported AEFIs were consistent with those reported in prior studies and the large majority were mild and self-limiting. These findings support current recommendations by WHO and the Thailand MOPHfor influenza vaccination of pregnant women. However, these vaccine recommendations are only impactful if they are implemented and accepted by healthcare providers and pregnant women themselves. Communication messages underscoring the safety of influenza vaccination for pregnant women therefore should be tailored to healthcare providers, and pregnant women to increase vaccination uptake. Indeed this is already being undertaken by the Thailand National Immunization Program. In the future, AEFI systems for pregnant women in Thailand should be strengthened to permit prospective and continuous collection of vaccine safety data and monitoring for rarer events.

## Abbreviations

AEFI: adverse event following immunization
ANC: antenatal care
CI: 95% confidence interval
IIV3: inactivated, trivalent influenza vaccine
IQR: interquartile range
MOPH: Ministry of Public Health
SAE: serious adverse event
WHO: World Health Organization
